# Personality Profile in Orthorexia Nervosa and Healthy Orthorexia

**DOI:** 10.3389/fpsyg.2021.710604

**Published:** 2021-09-14

**Authors:** María Roncero, Juan Ramón Barrada, Gemma García-Soriano, Verónica Guillén

**Affiliations:** ^1^Departamento de Personalidad, Evaluación y Tratamientos Psicológicos, Facultad de Psicología, Universitat de València, Valencia, Spain; ^2^Departamento de Psicología y Sociología, Universidad de Zaragoza, Teruel, Spain

**Keywords:** orthorexia nervosa, healthy orthorexia, personality profile, neuroticism, Disinhibition, psychoticism

## Abstract

Little is known about orthorexia in both its pathological (orthorexia nervosa, OrNe) and adaptive (healthy orthorexia, HeOr) forms. To date, few studies have been carried out to analyze the personality profile associated with orthorexia, and the results have been contradictory. The aim of the present study was to explore the characteristic pathological personality traits associated with OrNe and HeOr. A total of 297 participants (*M*_age_ = 30.8 years; 94.6% women) from the general population completed the Teruel Orthorexia Scale (TOS) and Personality Inventory for DSM-5-Short Form (PID-5-SF) questionnaires. Results showed significant low-medium correlations between OrNe and the four personality factors (Negative Affectivity, Detachment, Disinhibition, and Psychoticism) (*r*s range [0.08, 0.36]). In the regression analysis, the predictors of OrNe were Neuroticism and, to a lesser extent, Psychoticism. For HeOr, the associations were lower (*r*s range [−0.13, 0.05]) and negative, except Psychoticism. Only the relationship with Disinhibition was statistically significant, although after controlling for OrNe, Negative Affectivity and Antagonism also became significant. In the regression analysis, the predictors of HeOr were Disinhibition (negative direction) and Psychoticism (positive direction). The findings show that OrNe is associated with a pathological personality pattern characterized by difficulty in regulating emotions and negative affect (Negative Affectivity), as well as eccentricity, feeling special, and holding beliefs outside the norm (Psychoticism). However, HeOr seems to be related to the tendency toward high responsibility, self-control, the ability to maintain the focus of attention (low Disinhibition), and Psychoticism. Future studies should confirm whether this combination is a key component underlying the development and maintenance of orthorexia.

## Introduction

Orthorexia nervosa (OrNe) is characterized by excessive preoccupation with healthy eating that causes distress and interference due to extremely rigid self-imposed rules in relation to one's eating habits (Varga et al., 2014; Dunn and Bratman, 2016; Cena et al., 2019). People with high levels of OrNe are overly concerned about the quality of the food they eat in terms of its composition (food additives or preservatives, among others), origin, and processing. Surprisingly, there is little evidence that people with high levels of OrNe follow a healthier diet (Zickgraf and Barrada, 2021). Recently, the other side of the orthorexia coin was described, i.e., the adaptive version of the interest in healthy eating. This new dimension of orthorexia is called “healthy orthorexia” (HeOr), which can be defined as healthy interest in diet, healthy behavior with regard to diet, and eating healthily as part of one's identity (Barrada and Roncero, 2018; Depa et al., 2019). Importantly, HeOr is not a measure of healthy eating and, thus, should not be confused with it. People who score high on HeOr indicate that healthy eating is an important part of their life and that they devote time and energy to it. However, their beliefs about healthy eating may not coincide with an objective or real definition of healthy eating. For this reason, the two terms should not be taken as synonyms because a high score on HO does not necessarily mean that the person is eating healthily. The two dimensions of orthorexia present very different patterns of associations with different measures of psychopathology: whereas OrNe is positively associated with negative affect, disordered eating, and obsessive-compulsive symptoms, HeOr shows the opposite pattern of associations (Barrada and Roncero, 2018; Barthels et al., 2019; Depa et al., 2019; Strahler et al., 2020).

In the area of orthorexia, the majority of the research has focused on OrNe. Although psychiatric manuals do not consider it a mental disorder, different diagnostic criteria have been proposed. Several attempts have been made in this direction (Varga et al., 2013; Barthels et al., 2015a; Moroze et al., 2015; Dunn and Bratman, 2016), and, in general, the following criteria stand out: (a) excessive preoccupation with healthy eating, (b) negative emotional consequences, and (c) interference in relevant areas of life along with malnutrition and weight loss (Cena et al., 2019). Nevertheless, these criteria have been questioned. For instance, the association between OrNe and body mass index is not clear (McComb and Mills, 2019), and so it is not clear whether weight loss should be a diagnostic criterion. Studies mostly show a medium-high significant and positive relationship between OrNe and the symptomatology of ED and obsessive-compulsive disorder (OCD) (Koven and Abry, 2015; Moroze et al., 2015; Bundros et al., 2016; Barrada and Roncero, 2018; Bartel et al., 2020; Domingues and Carmo, 2021). However, despite the association between OrNe and ED symptomatology and the risk factors they share, data suggest that OrNe presents a dysfunctional pattern associated with eating that differs from the two main eating styles that characterize ED, dietary restraint and emotional eating, which justifies the need for further research on this condition (Barthels et al., 2019).

To shed light on the clinical description of OrNe, there is a line of research that analyzes its psychopathological correlates. With regard to personality traits, most definitions point to the relevance of certain traits, most of them associated with ED and OCD. There is a consensus about the importance of perfectionism (Bundros et al., 2016; Barrada and Roncero, 2018; Bartel et al., 2020), narcissism (Oberle et al., 2017), rigidity, need for control, and self-discipline (Koven and Abry, 2015; Domingues and Carmo, 2019; Cheshire et al., 2020).

Very few studies have been carried out on the general dimensions of personality, and some of them have used the ORTO questionnaire (Donini et al., 2005; Gramaglia et al., 2019; Kiss-Leizer and Rigó, 2019), a measure of OrNe that has raised serious validity concerns (Roncero et al., 2017; Opitz et al., 2020; Mitrofanova et al., 2021). In studies with valid measures, we can distinguish between two lines of research. The first personality model considers and assesses the Big Five model. As far as we know, two studies have used this approach with community samples from Germany and Lebanon (Strahler et al., 2020) and undergraduate students from the U.S. (Gleaves et al., 2013). The first study, using two different instruments to measure orthorexia—the Teruel Orthorexia Scale (TOS) (Barrada and Roncero, 2018) or the Düsseldorf Orthorexia Scale (Barthels et al., 2015b)—found inconsistent results, both in terms of the sample origin and the orthorexia measure. For instance, the association between Neuroticism and OrNe was statistically significant for both scales in the German sample, whereas it was weaker and only significant for the TOS scores in the Lebanese sample. For OrNe, of the 20 correlations, only three were over |0.20| (we use this arbitrary cut-off point to simplify the description of the previous results): negative associations with Neuroticism for both scales in the German sample; a negative association with Agreeableness in the Lebanese sample with the TOS. With regard to HeOr, of the 10 correlations, the only correlation over |0.20| was for Extraversion in the German sample. In the U.S. sample (Gleaves et al., 2013), and with the same cut-off point, only a single correlation was above |0.20|: the one between OrNe and Neuroticism.

The second way to conceptualize personality was proposed by the American Psychiatric Association (2013). This dimensional personality model, which mainly focuses on maladaptive traits, also encompasses five factors, several of which highly overlap with those from the Big Five model: Negative Affectivity (similar to Neuroticism in the Big Five model), Detachment (as opposed to Extraversion), Antagonism (as opposed to Agreeableness), Disinhibition (as opposed to Conscientiousness), and Psychoticism, with this latter dimension having no clear correspondence with any Big Five dimension. Strahler et al. (2020) also analyzed the associations between OrNe and HeOr and these maladaptive personality traits. For OrNe, only five correlations were over |0.20|, all of which corresponded to the German sample and the TOS scores. For HeOr, no correlations were above this cut-off point. In this study, the Personality Inventory for DSM-5-Brief Form (25 items) was used (Fossati et al., 2017).

After reviewing the available studies to date, given their scarcity and contradictory results, more studies are needed to determine the personality traits associated with OrNe and HeOr. We will focus on the maladaptive personality traits. Given that OrNe is associated with psychopathology, it has been proposed as a mental disorder, and few studies have used this approach. Improved knowledge about these associations, on the one hand, would help to understand their etiology, not only for the treatment of OrNe patients, but also for prevention and maintenance of patient recovery. On the other hand, it would shed some light on the nature of the HeOr, which has been found to show negative associations with clinical variables, whereas the relationship with personality traits remains unclear. Thus, the aim of this study was to explore the characteristic pathological traits associated with OrNe and HeOr. Based on previous studies, only two clear expectations could be defined. The first is to find a different pattern between the personality traits in OrNe and HeOr, and the second is to find positive associations between OrNe and pathological personality traits, mainly Negative Affectivity.

## Method

### Procedure and Participants

A convenience sample was used in the present study. People were invited to participate through advertisements placed in the Facebook and Instagram accounts of the authors of this study and students of the Bachelor and Master's Degrees in Psychology. These ads invited them to participate in a study on healthy eating habits and psychological variables by completing a survey that would take about 20 min. A link to the survey, which was hosted on the LimeSurvey platform, was added at the end of the post. On the first screen of this questionnaire, an informed consent form provided information about their participation in the study and contact details of the principal researcher. All participants had to agree to the informed consent in order to complete the questionnaires. Participants took part voluntarily in the study, and they received no compensation for their collaboration. Inclusion criteria to participate in the study were being over 18 years old and fluent in Spanish. This study was approved by the Ethics Commission of the University of Valencia (code H1543497880249).

A total of 297 participants completed the measures, 281 women (94.6%) and 16 men (5.4%). The mean age was 30.8 years (*SD* = 12.9, range [18, 65]). With regard to education, 10 (3.4%) of the sample had a primary level, 79 (26.6%) had a secondary level, and 208 (70.0%) had a tertiary level. Regarding relationship/marital status, 177 (59.6%) participants were single, 53 (17.8%) were not married and living with their partner, 51 (17.2%) were married, 15 (5.1%) were divorced/widow, and one person (0.3%) did not respond to this question.

### Instruments

#### Teruel Orthorexia Scale (TOS)

The TOS (Barrada and Roncero, 2018) is a self-report scale that assesses orthorexia in two separate dimensions, HeOr (nine items; e.g., “I mainly eat foods that I consider healthy”; Cronbach's α = 0.85 –all reported alphas correspond to values obtained with the current sample–) and OrNe (eight items; e.g., “Thoughts about healthy eating do not let me concentrate on other tasks”; α = 0.74). Responses are given on a 4-point scale ranging from 0 = *Completely disagree* to 3 = *Completely agree*. Scores for each dimension were computed as the sum of the item responses.

#### Personality Inventory for DSM-5-Short Form (PID-5-SF)

The PID-5-SF (Maples et al., 2015) is a self-report questionnaire that assesses the five maladaptive traits with 60 items, 12 per dimension. These dimensions are: Negative Affectivity (“I worry a lot about terrible things that might happen”; α = 0.87); Detachment (“I keep my distance from people”; α = 0.87); Antagonism (“I don't hesitate to cheat if it gets me ahead”; α = 0.84); Disinhibition (“I feel like I act totally on impulse”; α = 0.86); Psychoticism (“Others seem to think I'm quite odd or unusual”; α = 0.82). Responses are given on a 4-point scale ranging from 0 = *Very false or often false* to 3 = *Very true or often true*. Scores for each dimension were computed as the mean of the item responses. For the present study, the Spanish adaptation of the PID-5 was used (Díaz-Batanero et al., 2019).

### Data Analysis

We computed descriptive statistics (means, standard deviations, skewness, and kurtosis) and correlations between the two orthorexia dimensions and the five personality scores. We plotted the distribution of the two orthorexia dimensions. We present Pearson correlation sizes for all the bivariate associations, and compared the correlation sizes for each personality dimension and the two orthorexia factors (Hittner et al., 2003). We computed partial correlations for all the associations between orthorexia and personality scores while controlling for the other orthorexia dimension. Second, we computed linear regression models with the two orthorexia dimensions as criteria and the five personality dimensions as predictors. All the predictors were introduced at the same time. We report standardized coefficients for these regression models. The analyses were performed with R 4.0.4. The open database and code files for these analyses are available at the Open Science Framework repository (https://osf.io/k56pc/).

## Results

Table 1 shows the associations among the different variables with the descriptive statistics. The mean orthorexia scores were comparable to previous results (Barrada and Roncero, 2018; Depa et al., 2019), despite the important differences in the sample composition and participant recruitment method. For OrNe, in the present sample *M* (*SD*) = 3.69 (3.12), whereas in Barrada and Roncero (2018) *M* (*SD*) = 3.44 (3.57) and in Depa et al. (2019) *M* (*SD*) = 4.32 (4.05). For HeOr, in the present sample *M* (*SD*) = 13.45 (5.22), whereas in Barrada and Roncero (2018) *M* (*SD*) = 12.52 (5.22) and in Depa et al. (2019) *M* (*SD*) = 12.71 (5.26). All Cohen's *d*s of the different comparison were smaller than 0.18.

**Table 1:** Descriptive statistics and correlations between the different orthorexia and personality scores.

	**Descriptives**				**Pearson Correlations**							**Differences in Correlations**		**Partial Correlations**	
	***M***	***SD***	***Sk***	***K***	**NAF**	**DET**	**ANT**	**DIS**	**PSY**	**OrNe**	**HeOr**	***diff r***	***p diff***	***r*_**P**_**	***r*_**P**_**
PID Negative Affectivity	0.93	0.59	0.66	0.04		**0.37**	**0.26**	**0.59**	**0.44**	**0.36**	−0.05	**0.41**	** <0.001**	**0.39**	**−0.16**
PID Detachment	0.44	0.46	1.60	3.29			**0.20**	**0.46**	**0.48**	**0.23**	−0.02	**0.25**	** <0.001**	**0.25**	−0.09
PID Antagonism	0.35	0.39	1.34	1.17				**0.39**	**0.40**	0.08	−0.10	**0.17**	**0.012**	0.11	**−0.12**
PID Disinhibition	0.65	0.49	0.83	0.14					**0.62**	**0.21**	**−0.13**	**0.34**	** <0.001**	**0.26**	**−0.20**
PID Psychoticism	0.34	0.39	1.51	2.46						**0.26**	0.05	**0.21**	**0.002**	**0.26**	−0.03
TOS OrNe	3.69	3.12	1.07	0.88							**0.28**	-	-	-	-
TOS HeOr	13.45	5.22	0.00	−0.43								-	-	-	-

*PID, personality inventory for DSM-5-short form; NAF, negative affectivity; DET, detachment; ANT, antagonism; DIS, disinhibition; PSY, psychoticism; TOS, teruel orthorexia scale; OrNe, orthorexia nervosa; HeOr, healthy orthorexia; diff r corresponds to the difference r(personality dimension,OrNe)–r(personality dimension,HeOr); p diff corresponds to the statistical significance of that difference; r_p_, partial correlation controlling for the other orthorexia dimension. Bold values correspond to statistically significant associations and differences, p < 0.05*.

**Figure 1:**
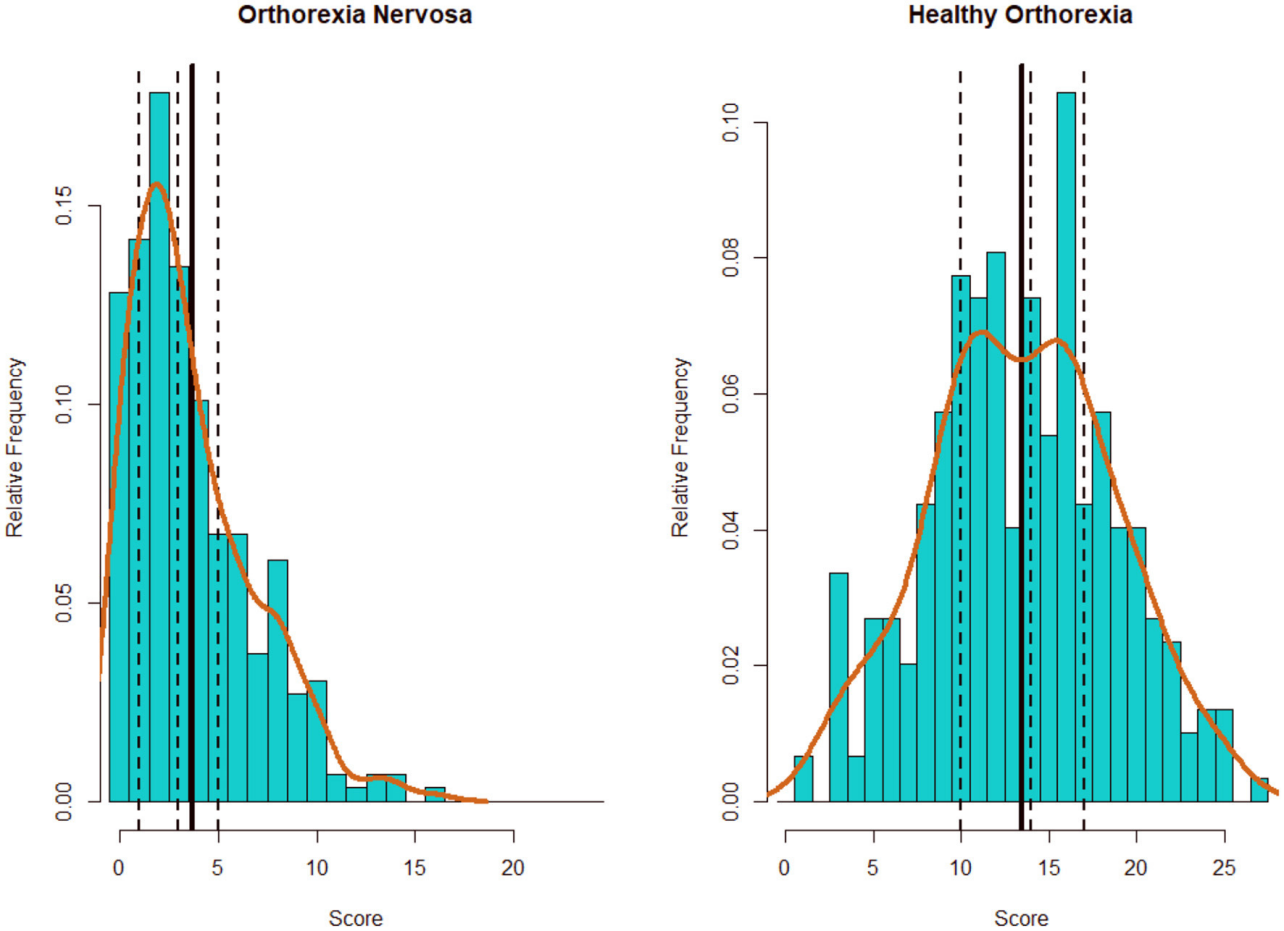
Distribution of the TOS scores by dimension. Solid vertical line corresponds to the mean value. Dashed vertical lines, from left to right, correspond to first, second (median), and third quartile. Brown line correspond to the density plot.

As would be expected, the mean values for the maladaptive personality dimensions were low. All the scores showed a positive skewness, except for HeOr. Detachment showed a higher kurtosis. The distribution of the two TOS scores can be seen in Figure 1. As would be expected, OrNe presented a positive skewness. HeOr was symmetrical. The five personality scores showed medium-high correlations (mean *r* = 0.42, range [0.20, 0.62]). The two dimensions of orthorexia showed a small-medium correlation, *r* = 0.28.

Focusing on the correlations between orthorexia and personality, all the Pearson correlations for OrNe were positive, low-medium (mean *r* = 0.23, range [0.08, 0.36]), and statistically significant (*p*s < 0.001), except Antagonism (*p* = 0.172). The highest correlation was with Negative Affectivity. This pattern was different for HeOr. For this orthorexia dimension, the correlations were smaller (mean |*r*| = 0.07, range of |r| [0.02, 0.13]) and negative, except Psychoticism, and only the correlation with Disinhibition was statistically significant (*p* = 0.031; the other *p*s ≥ 0.101). Importantly, all the sizes of the correlations between the personality scores and the two orthorexia dimensions showed statistically significant differences (*r*(personality dimension,OrNe)–*r*(personality dimension,HeOr)). The size of these differences ranged from 0.41 for Negative Affectivity to 0.17 for Antagonism, with all *p*s ≤ 0.012.

When controlling for the other orthorexia dimension, the results of the partial correlations for OrNe were almost equivalent to those for the Pearson correlations (mean *r* = 0.25, range [0.11, 0.39]), and they had the same statistically significant and non-significant values. In the case of HeOr, the partial correlations diverged to a greater degree. In this case, all the associations were negative, and four were larger and statistically significant (mean |*r*| = 0.12, range of |*r*| [0.03, 0.20]).

With regard to the regression analyses, the models for OrNe and HeOr were both statistically significant [*F*_(5,291)_ = 10.56, *p* < 0.001 for OrNe; *F*_(5,291)_ = 3.09, *p* = 0.010 for HeOr], although the explained variance was four times larger for OrNe ($R_{adj}^{2}$ = 0.139) than for HeOr ($R_{adj}^{2}$ = 0.034). These results are shown in Table 2. For OrNe, two personality dimension coefficients were statistically significant and positive, Negative Affectivity (beta = 0.32, 95% CI [0.19, 0.46], *p* < 0.001) and Psychoticism (beta = 0.16, 95% CI [0.01, 0.30], *p* = 0.033). For the other personality dimensions, the coefficients were smaller and had mixed signs, with all *p*s ≥ 0.157. A surprising pattern of results was found for HeOr. In this case, two regression coefficients were also statistically significant, although with different signs: negative for Disinhibition (beta = −0.24, 95% CI [−0.40, −0.07], *p* = 0.005) and positive for Psychoticism (beta = 0.24, 95% CI [0.08, 0.39], *p* = 0.003). For the other three personality dimensions, the coefficients were smaller and mixed in their signs, with all *p*s ≥ 0.117.

**Table 2:** Multiple regression analyses for the two orthorexia dimensions.

	**TOS Orthorexia Nervosa**			
	**beta [95% CI]**	**SE**	***t***	***p***
PID Negative Affectivity	**0.32 [0.19, 0.46]**	**0.07**	**4.81**	**<** **0.001**
PID Detachment	0.09 [−0.03, 0.22]	0.06	1.42	0.157
PID Antagonism	−0.05 [−0.17, 0.07]	0.06	−0.82	0.413
PID Disinhibition	−0.10 [−0.25, 0.06]	0.08	−1.25	0.213
PID Psychoticism	**0.16 [0.01, 0.30]**	**0.07**	**2.15**	**0.033**
	**TOS Healthy Orthorexia**			
PID Negative Affectivity	0.02 [−0.12, 0.16]	0.07	0.24	0.808
PID Detachment	−0.01 [−0.14, 0.12]	0.07	−0.16	0.877
PID Antagonism	−0.10 [−0.23, 0.03]	0.06	−1.57	0.117
PID Disinhibition	**−0.24 [−0.40**, **−0.07]**	**0.08**	**−2.85**	**0.005**
PID Psychoticism	**0.24 [0.08, 0.39]**	**0.08**	**3.02**	**0.003**

*TOS, teruel orthorexia scale; CI, confidence interval; PID, personality inventory for DSM-5-short form; SE, standard error. Bold values correspond to statistically significant coefficients, p < 0.05*.

## Discussion

The aim of the present study was to analyze the personality traits associated with OrNe and HeOr. As expected, and although OrNe and HeOr are positively correlated, our first results showed a different pattern of relationships of the personality dimensions with OrNe and HeOr. Whereas the associations found between pathological personality traits and OrNe were positive, those found with HeOr were mainly in a negative direction and smaller. These results are coherent with the fact that OrNe and HeOr are two separable factors related to healthy eating. Previous studies observed associations in opposite directions between OrNe and HeOr and different variables, such as negative affect, symptoms of obsessive-compulsive and eating disorders (positive association with OrNe and negative with HeOr) (Barrada and Roncero, 2018; Barthels et al., 2019; Depa et al., 2019), and the mindfulness trait (negative association with OrNe and positive with HeOr) (Strahler, 2021). Even the reasons for food choices have been found to be different in OrNe and HeOr (Depa et al., 2019).

Regarding the specific relationships found between the maladaptive personality factors and OrNe, our results reveal small and positive relationships with four of the five personality factors (except Antagonism). This result is coherent with the significant association between OrNe and the five PID factors found in the study with a German sample, although, surprisingly, in the Lebanese sample, the relationship with Negative Affectivity and Disinhibition was not significant (Strahler et al., 2020). When comparing the German, Lebanese, and present Spanish results, the results from Lebanon are the most divergent. The results of our regression analysis showed that two personality factors contributed to the prediction of OrNe: Negative Affectivity and Psychoticism. The highest coefficient was for Negative Affectivity. The relevance of the association between OrNe with Negative Affectivity (or the almost equivalent Neuroticism or Negative Affect) has been observed in previous studies. In the Gleaves et al. (2013) study, the Problems factor of the EHQ was mainly correlated with Neuroticism. This result was also replicated in the association found in the German sample (Strahler et al., 2020). Moreover, Negative Affect and OrNe have also been found to be related in other studies (Barrada and Roncero, 2018; Barthels et al., 2019). Thus, our results could suggest that people with a pathological preoccupation with eating healthily are more prone to anxiousness, emotional lability, and anger. With regard to the weight of the Psychoticism factor in the prediction of OrNe, this result is not surprising because it has been pointed out previously (Koven and Abry, 2015). It is consistent with the result in the German sample showing that Psychoticism was the personality trait with the highest association with OrNe (Strahler et al., 2020). Moreover, other studies have found magical ideation and/or erroneous beliefs related to food in orthorexia, symptoms that would fall within the psychotic spectrum (Lindeman et al., 2000). Additionally, Lasson and Raynal (2021) described a personality profile associated with OrNe, composed of high paranoid and narcissistic traits with moderate levels of schizotypal and borderline traits. Thus, the relationship with the psychotic spectrum could be explained by the feeling of being special and different from others and having beliefs and feelings different from the majority of people, which could be due to the lack of trust in others and the extravagance of systematically questioning the nature, origin, and safety of the food that the vast majority of the population consumes regularly. Indeed, people who are highly preoccupied with healthy eating are likely to be suspicious of products sold in most supermarkets, and they try to buy their food from places that seem safer to them, such as organic or local shops. In this regard, OrNe's characteristic concern about avoiding toxic or harmful food is one of the worries included in the concept of Modern Health Worries (MHWs). MHWs are defined as “perceived risk to personal health from technological changes and features of modern life” (Petrie et al., 2005). They include a factor called “tainted food,” which consists of concerns about foods with elements such as hormones, antibiotics, additives, pesticides, and genetically modified foods. People with high MHWs show a preference for natural foods rather than foods that include synthetic additives, and they are more accepting of foods that have been designed to reduce the risk of disease (Devcich et al., 2007). In fact, the MHW construct has been linked to a paranoid profile (Lahrach and Furnham, 2017; Dömötör et al., 2019).

Regarding the relationship between personality factors and HeOr, the associations were negative, except for Psychoticism, and small. In fact, only the correlation with Disinhibition reached statistical significance. When the effect of OrNe was controlled, the size of all the associations (still negative) increased, and two additional associations reached significance (Negative Affectivity and Antagonism). This result is in line with our hypothesis, that is, negative associations between pathological personality traits and the non-pathological preoccupation with healthy eating. The inverse relationship with negative affect is consistent with previous results showing an inverse relationship with negative affect when controlling for OrNe weight (e.g., Barrada and Roncero, 2018). These results also point to the relevance of considering the overlap between the two types of orthorexia because partial correlations and Pearson correlations may lead to different interpretations. Regression analyses provided noteworthy results. Statistically significant predictors were Disinhibition, in a negative direction, and Psychoticism, in a positive direction. The ability to predict HeOr by means of personality traits was very low. These results suggest that the person with an adaptive preoccupation with eating healthy would present the tendency to be responsible and self-controlled and the ability to focus his/her attention (low Disinhibition). In other words, people who are preoccupied with healthy eating, and for whom healthy eating is an important part of life, would be able to commit themselves to the goal of healthy eating, taking responsibility for their actions, persisting, and not getting carried away by secondary aspects such as the palatability of food. They would take care of themselves and others, especially with regard to food, and be able to plan and take into account the long-term consequences because food choices would be made on the basis of medium to long-term benefits (health) rather than immediate reinforcement (taste). Regarding the relationship between psychoticism and HeOr, we did not expect to find a positive relationship with any pathological personality trait. The results show that people with high HeOr scores have a tendency to be eccentric and hold unusual beliefs and experiences (Psychoticism). These people may show a certain distrust and suspicion of the intentions of others. This relationship could be explained in the same way as the relationship found with OrNe. Thus, people for whom healthy eating is a central aspect of their lives would have a tendency to feel special and different from other people, at least in the aspect of healthy eating that guides their lives. In fact, two of the items included in the HeOr factor are “I believe that the way I eat is healthier than that of most people” and “I try to convince people from my environment to follow my healthy eating habits.” In this regard, these individuals hold beliefs and experiences about eating “outside the norm” that are recognized by others as out of the ordinary, along the lines of MHWs (Petrie et al., 2005).

The present study has several limitations. First, the sample size is small, and so future studies should find out whether the same associations remain significant or whether any further associations emerge. Second, although the sample includes people from the general population, participants were invited by university students, and 94% are women, and so the sample is not representative of the general population. Third, the data were collected through self-reports administered through a web-based survey platform without checking for possible random responses, although the OrNe and HeOr levels are similar to those reported in previous studies.

Analyzing the results as a whole, it is remarkable to note that the pathological factor of interest in healthy eating (OrNe) is related to the difficulty in regulating emotions and negative affect. Negative Affectivity is a factor that is closely related conceptually and empirically to Neuroticism (Wright and Simms, 2014; Maples et al., 2015). This personality trait is considered a trans-diagnostic psychopathological substrate underlying numerous mental disorders, especially internalizing disorders (Samuels et al., 2000; Cervera et al., 2003; Lahey, 2009; Barlow et al., 2014), and it could be the basis for the characteristic psychopathology observed in OrNe, such as negative affectivity and eating and obsessive-compulsive disorder symptomatology, among others (Koven and Abry, 2015; Moroze et al., 2015; Bundros et al., 2016; Barrada and Roncero, 2018; Bartel et al., 2020; Domingues and Carmo, 2021). As far as the adaptive factor of orthorexia (HeOr) is concerned, a personality pattern is observed with a tendency toward responsibility, self-control, and the ability to maintain the focus of attention, accompanied by eccentricity, feeling special, and holding beliefs and experiences outside the norm. Future studies should confirm whether this combination of high neuroticism, low Disinhibition, and high psychoticism are key components underlying the development and maintenance of OrNe and HeOr, respectively. Moreover, given that the weight of personality traits does not seem to play a prominent role in explaining OrNe, future studies should examine the weight of other potentially relevant variables, such as metacognitive variables or other dysfunctional beliefs, that may underlie the genesis and maintenance of OrNe.

## Conclusion

The present study provides relevant data on the relationship between orthorexia, both the pathological (OrNe) and healthy (HeOr) aspects, and personality traits. To our knowledge, this is the first study conducted from the dimensional perspective of the DSM-5 personality traits using the PID-5-SF questionnaire (a previous study used the shorter version). Our results point to the relevance of three personality dimensions in orthorexia: (1) Negative Affectivity, which would be relevant for OrNe; (2) low Disinhibition, relevant for HeOr; and (3) Psychoticism, relevant for both dimensions of Orthorexia. Studies on personality traits associated with orthorexia, as in our case, help to shed light on the psychological profile of people who are preoccupied with healthy eating. This contributes to better understanding a construct that has scarcely been studied, although interest in it has increased exponentially in recent years at a social level, which in turn is reflected in the exponential increase in scientific studies being carried out in this field.

## Data Availability Statement

The datasets presented in this study can be found in online repositories. The open database and code files for these analyses are available at the Open Science Framework repository (https://osf.io/k56pc/).

## Ethics Statement

The studies involving human participants were reviewed and approved by Ethics Commission of the University of Valencia (code H1543497880249). The patients/participants provided their written informed consent to participate in this study.

## Author Contributions

MR, JB, GG-S, and VG contributed the conception and design of the study and wrote the second draft of the manuscript. MR carried out sample collection. MR and JB organized the database and wrote the first draft of the manuscript. JB performed the statistical analysis. All authors contributed to manuscript revision and read and approved the submitted version.

## Funding

This study was supported by the Conselleria d'Innovació, Universitats, Ciència i Societat Digital, Generalitat Valenciana (GV/2021/162), and by Ministry of Science and Innovation- State Research Agency of Spain, co-funded by the European Union European Regional Development Fund (Grant RTI2018-098349-B-I00).

## Conflict of Interest

The authors declare that the research was conducted in the absence of any commercial or financial relationships that could be construed as a potential conflict of interest.

## Publisher's Note

All claims expressed in this article are solely those of the authors and do not necessarily represent those of their affiliated organizations, or those of the publisher, the editors and the reviewers. Any product that may be evaluated in this article, or claim that may be made by its manufacturer, is not guaranteed or endorsed by the publisher.
